# Two kinds of transcription factors mediate chronic morphine-induced decrease in miR-105 in medial prefrontal cortex of rats

**DOI:** 10.1038/s41398-022-02222-3

**Published:** 2022-10-31

**Authors:** Junfang Zhang, Xinli Guo, Zhangyin Cai, Yan Pan, Hao Yang, Yali Fu, Zixuan Cao, Yaxian Wen, Chao Lei, Chenshan Chu, Yu Yuan, Dongyang Cui, Pengyu Gao, Bin Lai, Ping Zheng

**Affiliations:** 1 State Key Laboratory of Medical Neurobiology, MOE Frontiers Center for Brain Science, School of Basic Medical Sciences, Institutes of Brain Science, Fudan University, Shanghai, 200032 China; 2 Eye Institute, Affiliated Hospital of Nantong University, Nantong, 226001 China; 3 Department of Neurology of Zhongshan Hospital, Fudan University, Shanghai, 200032 China; 4 Medical College of China Three Gorges University, Yichang, 443002 China

**Keywords:** Molecular neuroscience, Epigenetics in the nervous system

## Abstract

Chronic morphine administration alters gene expression in different brain regions, an effect which may contribute to plastic changes associated with addictive behavior. This change in gene expression is most possibly mediated by addictive drug-induced epigenetic remodeling of gene expression programs. Our previous studies showed that chronic morphine-induced decrease of miR-105 in the medial prefrontal cortex (mPFC) contributed to context-induced retrieval of morphine withdrawal memory. However, how chronic morphine treatment decreases miR-105 in the mPFC still remains unknown. The present study shows that chronic morphine induces addiction-related change in miR-105 in the mPFC via two kinds of transcription factors: the first transcription factor is CREB activated by mu receptors-ERK-p90RSK signaling pathway and the second transcription factor is glucocorticoid receptor (GR), which as a negative transcription factor, mediates chronic morphine-induced decrease in miR-105 in the mPFC of rats.

## Introduction

Drug addiction is a chronic brain disorder characterized by compulsive repeated use of drugs [1]. Chronic exposure to addictive drugs, such as morphine, causes long-term changes in different brain regions called “addictive brain”, leading vulnerable individuals to engage in pathological drug seeking and drug taking that can remain a lifelong struggle [2]. Therefore, the study of the mechanism underlying addictive drug-induced long-term changes in the brain is of importance for developing new therapeutic approaches to prevent drug addiction.

Previous studies have found that chronic morphine administration alters gene expression in different brain regions, which may contribute to plastic changes associated with addictive behavior [3–6]. This change in gene expression is most possibly mediated by addictive drug-induced epigenetic remodeling of gene expression programs, which alters gene expression without a change in nucleotide sequence of DNA [7], in discrete brain regions. Moreover, the kinds of involved genes and the brain regions where the genes lie are closely related to addiction. For example, Ferguson et al. found that chronic morphine treatment selectively upregulated SIRT1, a kind of deacetylases that targeted histone in the nucleus accumbens (NAc) without altering other sirtuins. Overexpression of SIRT1 within the NAc potentiated morphine-induced retrieval of reward memory, whereas the knockdown of SIRT1 had an opposite effect [8]. The studies from our lab showed that (1) chronic morphine-induced increases in the expression of D1 receptors at presynaptic terminals coming from other structures to the basolateral amygdala (BLA) played an important role in conditioned context-induced retrieval of morphine withdrawal memory [9]; (2) the downregulation of miR-105 in neurons projecting from the mPFC to the BLA was the reason for chronic morphine-induced increases in the expression of D1 receptors at presynaptic terminals coming from mPFC to BLA and the overexpression of miR-105 in the mPFC inhibited conditioned context-induced retrieval of morphine withdrawal memory [10], suggesting that chronic morphine-induced decrease of miR-105 in the mPFC contributed to context-induced retrieval of morphine withdrawal memory. However, how chronic morphine treatment decreases miR-105 in the mPFC remains still unknown. In this paper, using western blotting, real-time PCR, RNAi technology, and behavioral assay method, we studied intracellular molecular mechanism underlying chronic morphine-induced decrease in the expression of miR-105 in the mPFC and its functional significance in conditioned context-induced retrieval of morphine withdrawal memory.

## Materials and methods

### Animals and chronic morphine treatment

Male Sprague-Dawley (SD) rats (220–250 g) were housed under a 12:12 h light/dark cycle in a temperature- and humidity-controlled environment with free access to food and water. All experimental procedures conformed to the guidelines of the Institutional Animal Care and Use Committee of Fudan University, Shanghai Medical College. The morphine dependence of rat was induced according to previously described procedures [10, 11]. Briefly, rats were intraperitoneal injected by an increment dose of morphine twice daily at 08.00 h and 19.00 h, and morphine doses were progressively increased from 10 mg/kg to 40 mg/kg (2× 10 mg/kg on day 1; 2× 20 mg/kg on day 2; 2× 30 mg/kg on day 3; and 2× 40 mg/kg on days 4 and 5). Control rats were treated with saline following the same procedure. The rats were sacrificed 2 h after the last time of morphine administration. At this time, morphine should be on board because there were reports that there was no significant decline of morphine concentration in plasma at 2 h after s.c. injection of morphine [12].

### Primary culture of rat mPFC neurons and drug treatment

The mPFC neurons were isolated and cultured as described previously [9, 13]. Briefly, newborn (0–1 d) SD rats were euthanized by decapitation. The mPFC was dissected and dissociated with 0.125% trypsin at 37 °C for 15 min; digestion was terminated by adding DMEM containing 20% fetal bovine serum (FBS). Tissue was puffed, filtered, centrifuged, and the cell pellets collected. The cells were then resuspended in complete culture medium and plated in 6-well culture plates coated with poly-D-lysine (100 µg/mL; Sigma) at a density of 2 × 10^6^ cells/well. The cells were incubated in DMEM supplemented with penicillin (100 U/mL) and streptomycin (100 U/mL). After 4 h, the medium was replaced with a neurobasal medium (Gibco, Auckland, New Zealand) supplemented with 2% B27 (Gibco) and 0.5 mM glutamine (Gibco). A cytosine arabinoside solution (0.5 μM) was added to the culture 24 h after plating to minimize glial cell proliferation. During the maintenance phase, half the medium in each well was replaced with fresh medium every 2 days. Five-day-old mPFC neurons were subjected to morphine treatment at the indicated times. Primary cultured mPFC neurons were pretreated with naloxone (10 μM), U0126 (10 μM), and LJI308 (1 μM) for 3 h followed by 72 h morphine (10 μM) treatment. The primary cultured mPFC neurons were collected 2 h after the last morphine administration. The nonselective opioid receptor antagonist naloxone was purchased from Sigma. The ERK1/2 inhibitor U0126 and the p90RSK inhibitor LJI308 were obtained from Selleck.

### Knockdown of GR

GR knockdown was achieved using lentiviral RNAi technology (GeneChem, Shanghai, China). For GR knockdown, a sequence targeting *GR* mRNA was inserted into the GV248 vector (LV-GR-RNAi). The *GR* mRNA target sequence was 5′-GGTCTGAAGAGCCAAGAGTTA-3′. To generate the negative control lentivirus (LV-NC-RNAi), the sequence 5′-TTCTCCGAACGTGTCACGT-3′ was inserted into the GV248 vector. LV-GR-RNAi and LV-NC-RNAi were transfected into primary cultured mPFC neurons for 48 h before morphine treatment.

### Animal surgery

Male SD rats (220–250 g) were anesthetized with ketamine and xylazine (160 mg/kg and 2 mg/kg body weight, respectively) and secured in a stereotaxic instrument (Stoelting). Microinjections were performed using needles connected to a 1-μl microsyringe (Hamilton) by polyethylene tubing and controlled by a syringe pump (Harvard Apparatus). The intended stereotaxic coordinates for the mPFC were as follows: AP, +3.2 mm; ML, ±0.8 mm; DV, −3.8 mm. Rats were injected with LV-GR-RNAi and LV-NC-RNAi bilaterally into the mPFC in a 1 μl volume within 10 min. After the injection, the needles were retained in place for an additional 10 min to allow the diffusion of the virus. The rats were allowed at least 7 days to recover before the behavioral experiment and the efficiency of virus was verified in mPFC slices by analyzing the expression of EGFP.

### Quantitative real-time PCR

Total RNA, including mRNA and microRNA (miRNA), was extracted from cells and tissue using the miRcute miRNA Isolation Kit (Tiangen, Shanghai, China) according to the manufacturer’s manual. Reverse-transcription was performed with FastKing gDNA Dispelling RT SuperMix (Tiangen). Quantitative real-time PCR (qPCR) analysis of pri-miR-105, and GR levels were performed with a SuperReal PreMix Plus (SYBR Green) using 40 cycles of amplification (95 °C for 10 s, 60 °C for 25 s, and 72 °C for 20 s). MiR-105 was reverse-transcribed using a microRNA first-strand cDNA synthesis kit from Tiangen. qPCR was performed on a Mastercycler ep realplex Real-time PCR System of eppendorf (40 cycles of amplification: 94 °C for 2 min, 94 °C for 20 s, and 60 °C for 34 s) using a miRcute microRNA qPCR detection kit (Tiangen). The primers for miR-105, pri-miR-105, and U6 were synthesized by Tiangen. Those for GR (RQP048912), and Gapdh (RQP049537) were purchased from GeneCopoeia (Guangzhou, China). To obtain the fold-change in mRNA and miRNA levels, the data were analyzed using the 2^–ΔΔCT^ method. The final gene expression levels were normalized to that of Gapdh for mRNA and U6 for miRNA. Triplicate reactions were carried out in three separate experiments.

### Western blotting

Total protein was extracted from cells and brain tissue using cold RIPA lysis buffer (50 mM Tris-HCl, pH 7.4, 150 mM NaCl, 1% NP-40, 0.5% sodium deoxycholate, 0.1% SDS, and 1% protease inhibitor), kept on ice for 30 min, and then centrifuged at 12,000 rpm for 10 min at 4 °C. The concentration of the protein extraction was quantified using a BCA kit (Pierce Chemicals). Equal amounts of protein were loaded and separated by SDS–PAGE and transferred to nitrocellulose membranes. After blocking in nonfat milk, the membranes were incubated with the following primary antibodies: rabbit polyclonal anti-GR (1:800, Abcam, ab3579) and mouse monoclonal anti-GAPDH (1:3000, Abclonal, AC002); and rabbit polyclonal anti-ERK (1:1000, Cell Signaling Technology, 4695), rabbit polyclonal anti-p-ERK (1:1000, Cell Signaling Technology, 4370), rabbit polyclonal anti-p90RSK (1:500, Cell Signaling Technology, 9355), rabbit polyclonal anti-pp90RSK (1:500, Cell Signaling Technology, 9341), rabbit polyclonal anti-CREB (1:1000, Cell Signaling Technology, 9197), and rabbit polyclonal anti-p-CREB (1:1000, Cell Signaling Technology, 9198). HRP-conjugated goat anti-mouse/rabbit IgG (1:10,000, LI-COR Bioscience, 925-32210 and 926-68071) were used as the secondary antibodies. The signals were detected using an Odyssey infrared imaging system (LI-COR Bioscience). Densities were quantified using Image J and normalized to that of GAPDH, which served as loading control. Each experiment was repeated at least three times.

### Conditioned place aversion

Conditioned place aversion (CPA) was assessed using a three-chamber (15 × 15 × 20 cm) apparatus (Med Associates, USA) with distinct visual and tactile environments to maximize contextual differences. The procedure for CPA was similar to that previously described [14]. First, rats were given a pre-test and allowed to freely explore the entire apparatus for 15 min. Rats with a strong unconditioned preference (>75% of the session time) for any compartment were excluded from the study. Microinjections of LV-GR-RNAi or LV-NC-RNAi into the mPFC were performed after the pre-test. After injection, the rats were allowed to recover for at least 7 days before the administration of chronic morphine treatment. Morphine dependence was induced in the animals by repeated intraperitoneal injections of morphine twice daily at 08.00 h and 19.00 h for 5 days, as described above. On days 6 and 8, 2 h after the administration of 40 mg/kg morphine, each rat was confined in its morphine withdrawal-paired compartment for 20 min immediately after an intraperitoneal injection of naloxone (0.1 mg/kg). On alternating days 7 and 9, two hours after the administration of 40 mg/kg morphine, the rats were confined in the opposite compartment (saline-paired compartment) for 20 min immediately after an intraperitoneal injection of saline. The post-test was conducted 24 h after conditioning on day 9; the rats were allowed to freely explore the three compartments for 15 min and the CPA score was calculated as the time in the morphine withdrawal-paired compartment minus the time in the saline-paired compartment.

### Statistical method

All data were analyzed using GraphPad Prism 8. Numerical data were expressed as means ± SEM. For two-group comparisons, two-tailed unpaired Student’s *t*-tests were used. For comparisons among multiple groups, analyses of variance (ANOVAs) were used. One-way ANOVA and two-way ANOVA were followed by Bonferroni’s post hoc test. *P*-value <0.05 was considered statistically significant.

## Results

### Chronic morphine downregulates the expression of miR-105 in the mPFC of rats, but acute morphine does not

**Fig. 1:**
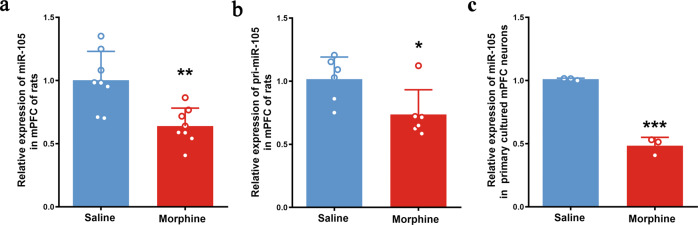
The effect of chronic morphine on the expressions of miR-105 and pri-miR-105 in rat mPFC and primary cultured mPFC neurons. **a** The relative expression of miR-105 in rats mPFC of saline and morphine groups (*n* = 8 in each group, unpaired two-tailed Student’s *t*-test, **P = 0.0019). **b** The relative expression of pri-miR-105 in rats mPFC of saline and morphine groups (*n* = 6 in each group, unpaired two-tailed Student’s *t*-test, *P = 0.0268). **c** The relative expression of miR-105 in primary cultured mPFC neuron of saline and morphine groups (*n* = 3 in each group, unpaired two-tailed Student’s *t*-test, ***P = 0.0002). Data are presented as mean ± SEM.

Our previous studies showed that chronic morphine downregulated miR-105 expression in the mPFC of rats [10]. To confirm this statement, here we repeated this experiment. The rats were randomly divided into two groups: saline group and morphine group. The level of miR-105 was detected by qPCR in the mPFC of rats treated with saline or morphine for 5 days. A significant decrease in miR-105 level was observed in morphine group (0.639 ± 0.050, *n* = 8, unpaired Student’s *t*-tests, *t* = 3.804, *P* = 0.0019), compared with saline group (1.00 ± 0.081, *n* = 8) (Fig. 1a). The miRNAs were transcribed as part of primary miRNAs (pri-miRNAs). To study whether chronic morphine-induced decrease in miR-105 was due to a decrease of primary miR-105 (pri-miR-105), we examined the influence of chronic morphine on pri-miR-105 expression by qPCR. Result showed that chronic morphine induced a significant decrease of pri-miR-105 level in the mPFC of rats (0.736 ± 0.080, *n* = 6, unpaired Student’s *t*-tests, *t* = 2.593, *P* = 0.0268), compared with saline group (1.015 ± 0.072, *n* = 6) (Fig. 1b). We also tested the effect of chronic morphine on the expression of miR-105 in primary cultured mPFC neurons of rats. Neurons were divided into two groups, saline group treated with saline for 3 days and morphine group treated with morphine (10 μM) for 3 days. The result showed that the miR-105 level significantly decreased after chronic morphine treatment (0.484 ± 0.038, *n* = 3, unpaired Student’s *t*-tests, *t* = 13.64, *P* < 0.0001), compared with saline group (1.010 ± 0.009, *n* = 3) (Fig. 1c). These results suggest that chronic morphine indeed downregulates the expression of miR-105 in the mPFC of rats. This statement is consistent with that of our previous report [10].

We also examined the effect of acute morphine on the expression of miR-105 in the mPFC of rats and in primary cultured mPFC neurons of rats. The result showed no significant change in the expression of miR-105 in the mPFC of rats at 2 h after one dose morphine (10 mg/kg) injection (1.066 ± 0.078, *n* = 4, unpaired Student’s *t*-tests, *t* = 0.1165, *P* > 0.05), compared with saline group (1.044 ± 0.173, *n* = 4) (Supplementary data Fig. 1a). Moreover, 2 h morphine treatment (10 μM) also had no significant effect on the expression of miR-105 in primary cultured mPFC neurons of rats (0.749 ± 0.183, *n* = 3, unpaired Student’s *t*-tests, *t* = 1.089, *P* > 0.05), compared with saline group (1.029 ± 0.181, *n* = 3) (Supplementary data Fig. 1b). These results suggest that acute morphine has no significant influence on the expression of miR-105 in the mPFC of rats.

### CREB activated by mu receptors-ERK-p90RSK signaling pathway is the first transcription factor that mediates chronic morphine induced-decrease in the expression of miR-105 in the mPFC

**Fig. 2:**
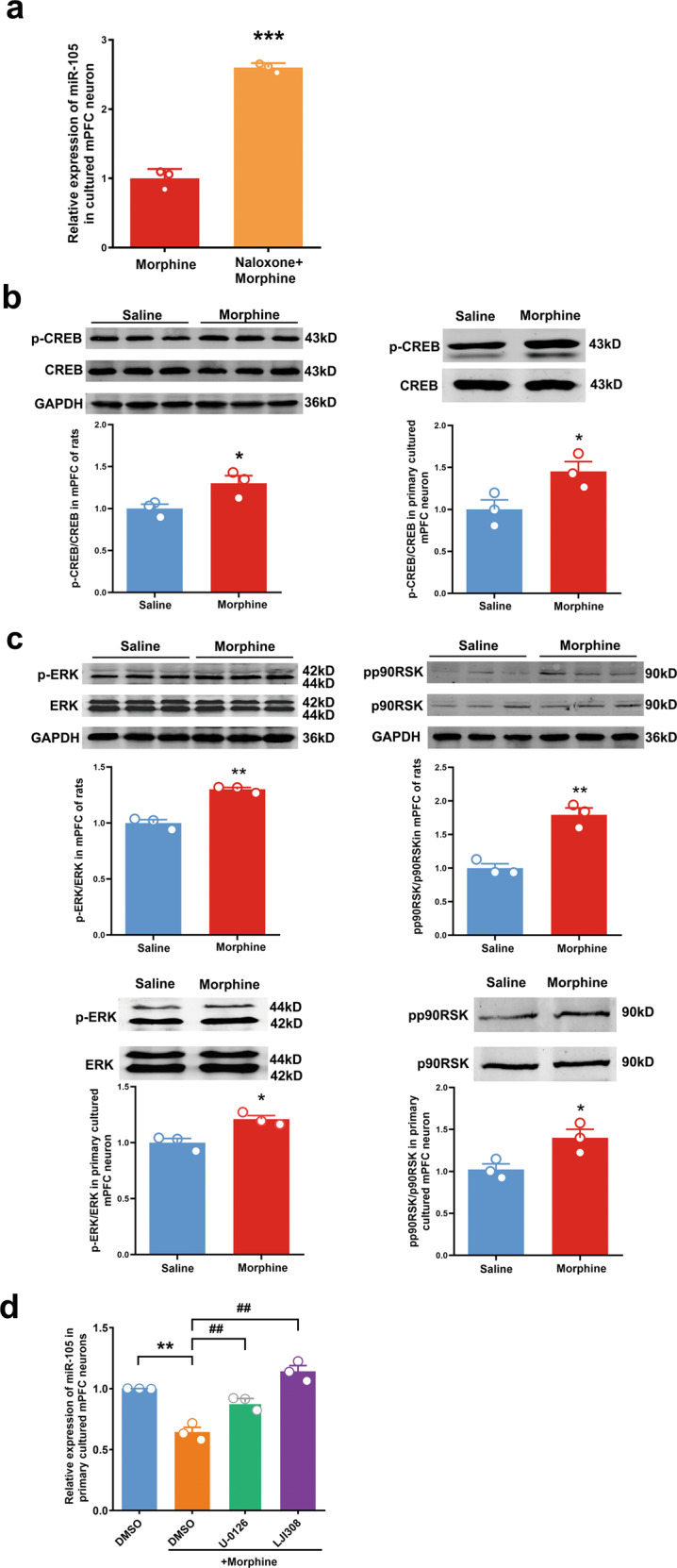
The influence of chronic morphine on the signaling molecules of CREB, ERK, and p90RSK in rat mPFC. **a** The influence of naloxone on the miR-105 expression in primary cultured mPFC neuron treated by morphine (*n* = 3 in each group, unpaired two-tailed Student’s *t*-test, ***P < 0.0001). **b** The expressions of p-CREB to total CREB in rat mPFC (left panel) and primary cultured mPFC neurons (right panel) (*n* = 3 in each group, unpaired two-tailed Student’s *t*-test, *P < 0.05, **P < 0.01). **c** The expressions of p-ERK/ERK and pp90RSK/p90RSK in rat mPFC and primary cultured mPFC neurons (*n* = 3 in each group, two-tailed Student’s *t*-test, *P < 0.05). **d** The expression of miR-105 in primary cultured mPFC neurons of DMSO group, DMSO + morphine group; U0126 + morphine group and LJI308 + morphine group (*n* = 3 in each group, one-way ANOVA, *F*_(3,8)_ = 37.40, *P* < 0.0001. Bonferroni post hoc: **P = 0.0005, DMSO vs DMSO + Morphine, ^##^*P* = 0.006 and 0.000, Morphine+U0126 and Morphine+LJI308 vs Morphine group, respectively). Data are presented as mean ± SEM.

It has been known that morphine is an agonist of opiate receptors. As the original site acted by morphine, opiate receptors should play a key role in chronic morphine-induced change in the expression of miR-105 in the mPFC of rats. To confirm this statement, we examined the influence of opiate receptor antagonist naloxone on chronic morphine-induced change in miR-105 in primary cultured mPFC neurons. Neurons were divided into two groups: morphine group treated with saline and morphine (10 μM); morphine + naloxone group treated with naloxone (10 μM) and morphine (10 μM). The result showed that after naloxone treatment, the change in miR-105 induced by chronic morphine disappeared (2.599 ± 0.038, *n* = 3, unpaired Student’s *t*-tests, *t* = 18.26, *P* < 0.0001), compared with morphine group (1.000 ± 0.079, *n* = 3) (Fig. 2a). We also examined whether naloxone alone had an influence on chronic morphine-induced change in the expression of miR-105. The result showed that naloxone alone had no a significant effect on the expression of miR-105 in primary cultured mPFC neurons (1.589 ± 0.161, *n* = 3, unpaired Student’s *t*-tests, *t* = 2.315, *P* = 0.082), compared with control group (1.029 ± 0.181, *n* = 3) (Supplementary data Fig. 2). This result suggests that opiate receptors may mediate chronic morphine-induced decrease in the expression of miR-105 in the mPFC in rats.

Opiate receptors mainly include three classes: the delta, kappa, and mu receptors [15]. Among them, morphine has a high selectivity for mu receptor [15]. Upon the activation by morphine, mu receptors are known to experience conformational changes, subsequently leading to different corresponding signaling pathways. Hui Zheng et al. proposed that the continued activation of Gi by mu receptor led to ERK phosphorylation, which then induced the subsequent activation of p90RSK and CREB [16]. To study the role of CREB in the change of miR-105 expression after chronic morphine treatment, the phosphorylation of CREB (p-CREB) was detected by western blotting. Rats and primary cultured mPFC neurons were divided into two groups: saline group and morphine group. The results showed that chronic morphine treatment could activate CREB in both the mPFC of rats (1.302 ± 0.0912, *n* = 3, unpaired Student’s *t*-tests, *t* = 2.869, *P* = 0.0455) and primary cultured mPFC neurons (1.453 ± 0.1164, *n* = 3, unpaired Student’s *t*-tests, *t* = 2.792, *P* = 0.0492), compared with the control group (Fig. 2b). We also examined the influence of chronic morphine on the phosphorylation of ERK1/2 and p90RSK. The result showed that chronic morphine increased the expression of p-ERK and pp90RSK in both the mPFC of rats (1.301 ± 0.0166, *n* = 3, *P* = 0.0009 and 1.794 ± 0.1008, *n* = 3, *P* = 0.0027) and primary cultured mPFC neurons (1.21 ± 0.0326, *n* = 3, *P* = 0.0136 and 1.40 ± 0.1021, *n* = 3. *P* = 0.0365) (Fig. 2c). Moreover, the pretreatment with U0126 (ERK1/2 inhibitor, 10 μM) or LJI308 (p90RSK inhibitor, 1 μM) blocked chronic morphine-induced decrease in the expression of miR-105 in primary cultured mPFC neurons (U0126: 0.8873 ± 0.0319, *n* = 3, *P* < 0.001; LJI308: 1.143 ± 0.0466, *n* = 3, *P* = 0.006, compared with morphine group (0.6436 ± 0.0396, *n* = 3) (Fig. 2d). These results suggest that CREB activated by mu receptors-ERK-p90RSK signaling pathway may be the first transcription factor that mediates chronic morphine induced-decrease in the expression of miR-105 in the mPFC.

### GR is the second transcription factor that mediates chronic morphine induced-decrease in the expression of miR-105 in the mPFC

**Fig. 3:**
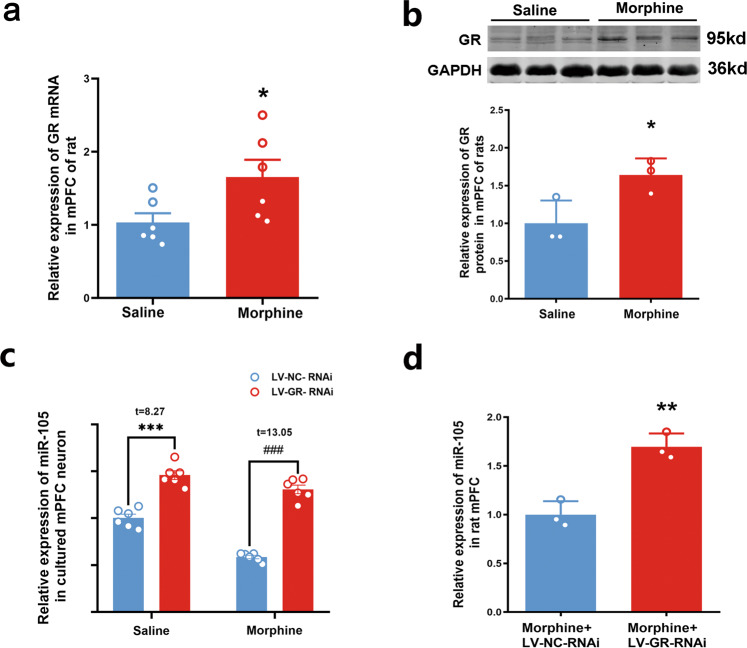
The expression of GR after chronic morphine treatment and its role on miR-105 expression. **a** GR mRNA level in rat mPFC in morphine and saline groups (*n* = 6 in each group, two-tailed *t*-test, *P = 0.0442). **b** GR protein level in rat mPFC in morphine and saline groups (*n* = 3 in each group, two-tailed *t*-test, *P = 0.0419). **c** The levels of miR-105 of primary cultured mPFC neurons treated by chronic morphine and LV-NC-RNAi or LV-GR-RNAi (*n* = 6 in each group, two-way ANOVA, *F*_(1,20)_ = 11.43, *P* = 0.003. Bonferroni’s post-tests: ***P < 0.0001, *t* = 8.270, saline+ LV-GR-RNAi group vs saline+ LV-NC-RNAi group; ^###^*P* < 0.0001, *t* = 13.050, morphine + LV-GR-RNAi group vs morphine + LV-NC-RNAi group). **d** The miR-105 levels in rat mPFC of morphine+ LV-GR-RNAi group and morphine + LV-NC-RNAi group (*n* = 3 in each group, unpaired two-tailed Student’s *t*-test, *t* = 6.185, **P = 0.0035). Data are presented as mean ± SEM.

GR is another important transcription factor [17]. In addition to the activation of the transcription of genes, GR is also able to repress the transcription of genes [18]. The binding of GR to GRE, a specific DNA motif, can suppress the expression of genes. Surjit et al. characterized the direct binding and repression of GR to widespread gene loci and reported that negative GREs were present in over 1000 mouse and human gene loci [19]. Therefore, it is possible that GR, as a negative transcription factor, mediates chronic morphine-induced decrease in miR-105 in the mPFC of rats. To test this hypothesis, first, we examined the influence of chronic morphine on the expression of GR mRNA and GR protein. The rats were randomly divided into two groups: saline group and morphine group. The level of GR mRNA and protein were detected by qRT-PCR and western blotting. The results showed that GR mRNA and protein level were significantly upregulated to 1.653 ± 0.2382 (*n* = 6, unpaired two-tailed Student’s *t*-test, *t* = 2.3, *P* = 0.0442) and 1.639 ± 0.2211 (*n* = 3, unpaired two-tailed Student’s *t*-test, *t* = 2.953, *P* = 0.0419) in morphine group, compared with that in saline group (GR mRNA, *n* = 6, 1.034 ± 0.1245 and GR protein, *n* = 3, 1.00 ± 0.1747) (Fig. 3a, b). Then, we examined the influence of the inhibition of GR using RNAi technology on chronic morphine-induced decrease in the expression of miR-105 in the mPFC of rats and in primary cultured mPFC neurons of rats. The neurons were divided into four groups: saline + LV-NC-RNAi group, saline + LV-GR-RNAi group, morphine + LV-NC-RNAi group, and morphine + LV-GR-RNAi group. Two-way ANOVA showed a statistically significant interaction between the effects of morphine and RNAi treatment on miR-105 expression (morphine × RNAi: *F*_(1,20)_ = 11.43, *P* = 0.003; morphine factor: *F*_(1,20)_ = 52.75, *P* < 0.0001; RNAi factor: *F*_(1,20)_ = 227.3, *P* < 0.0001. Bonferroni’s post-tests: saline + LV-NC-RNAi group vs. saline + LV-GR-RNAi group, *P* < 0.0001, *t* = 8.27; morphine + LV-NC-RNAi group vs. morphine + LV-GR-RNAi group, *P* < 0.0001, *t* = 13.050; saline + LV-GR-RNAi group vs morphine + LV-GR-RNAi group, *P* = 0.0749, *t* = 2.745, *n* = 6 in each group). This result indicated that the inhibition of GR expression increased miR-105 expression both in saline + LV-GR-RNAi and morphine + LV-GR-RNAi group, but the increase in miR-105 expression after the inhibition of GR from morphine + LV-NC-RNAi group to morphine + LV-GR-RNAi group was much higher than that from saline + LV-NC-RNAi group to saline + LV-GR-RNAi group (Fig. 3c). Moreover, there was a statistically significant interaction between the effects of morphine and RNAi treatment on miR-105 expression. Therefore, although under basal conditions, GR had some extent of inhibitory control on miR-105 expression, morphine-induced GR increase might have a much stronger inhibitory effect on miR-105 expression under chronic morphine treatment. We also studied the effect of GR inhibition by RNAi on miR-105 expression in the rats treated by chronic morphine. The rats were divided into two groups: morphine + LV-NC-RNAi group and morphine + LV-GR-RNAi group. The rats were injected with LV-GR-RNAi or LV-NC-RNAi as control into the mPFC of rats 7 days before chronic morphine treatment. The results showed that GR inhibition led to a significant increase in the level of miR-105 (1.695 ± 0.080, *n* = 3, unpaired two-tailed Student’s *t*-test, *t* = 6.185, *P* = 0.0035) in morphine + LV-GR-RNAi group, compared with that in morphine + LV-NC-RNAi group (1.000 ± 0.079, *n* = 3) (Fig. 3d).

**Fig. 4:**
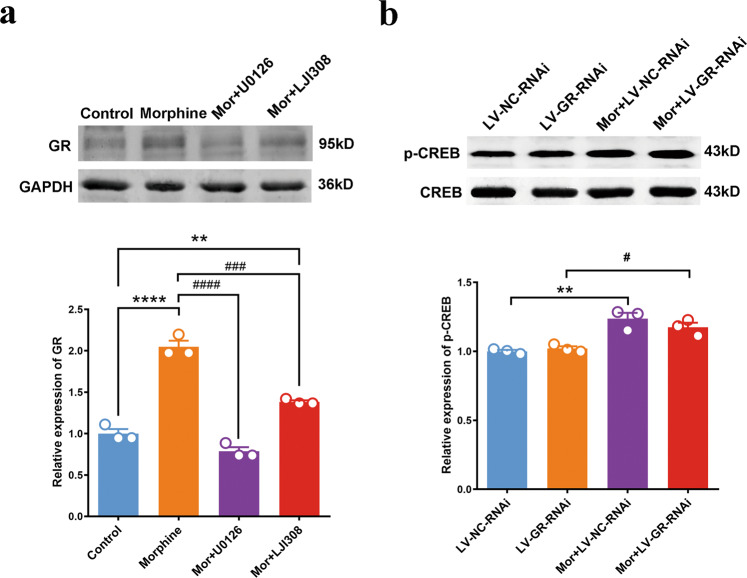
The role of ERK-p90RSK-CREB signaling pathway in GR expression increase induced by chronic morphine treatment in primary cultured neurons. **a** The levels of GR protein expression in primary cultured mPFC neurons of each group (*n* = 3 in each group, one-way ANOVA, *F*_(3,8)_ = 107.7, *P* = 0.000, Bonferroni post hoc: **P = 0.031, Morphine group vs Control group ^##^*P* = 0.000, Mor+U0126 group and Mor+LJI308 group vs Morphine group). **b** The level of p-CREB in primary cultured mPFC neurons of each group (*n* = 3 in each group, one-way ANOVA, *F*_(3,8)_ = 16.37, *P* = 0.009, Bonferroni post hoc: **P = 0.002, Mor+LV-NC-RNAi group vs LV-NC-RNAi group; ^#^*P* = 0.032, Mor+LV-GR-RNAi group vs LV-GR-RNAi group; *P* = 0.097, Mor+LV-NC-RNAi group and Mor+LV-GR-RNAi group). Data are presented as mean ± SEM.

Next, we explored the possible upstream mechanism underlying chronic morphine-induced increase in the expression of GR in the mPFC of rats. Based on the above results, CREB activated by mu receptors-ERK-p90RSK signaling pathway is the first transcription factor that mediates chronic morphine induced-decrease in the expression of miR-105 in the mPFC of rats. Therefore, we propose a hypothesis that this signaling pathway may be an upstream mechanism underlying chronic morphine-induced increase in GR expression in the mPFC of rats. To test this hypothesis, we examined the influence of the inhibition of ERK or p90RSK using the ERK inhibitor U0126 [20] or the p90RSK inhibitor LJI308 [21] on chronic morphine-induced increase of GR in primary cultured mPFC neurons. Neurons were divided into four groups: saline + DMSO group, morphine + DMSO group, U0126 + morphine group, and LJI308 + morphine group. GR protein levels in primary cultured mPFC neurons were subsequently detected by Western blotting. The result showed that morphine treatment increased GR protein level from 1.000 ± 0.05476 in saline + DMSO group to 2.048 ± 0.0746 in morphine + DMSO group (*n* = 3, *P* = 0.031, saline + DMSO group vs morphine + DMSO group, one-way ANOVA), but in the presence of U0126 or LJ1308, morphine-induced increase in GR protein level was decreased to 0.7853 ± 0.050 in U0126 + morphine group (*n* = 3, *P* < 0.0001, compared with morphine group, one-way ANOVA) or to 1.383 ± 0.0184 in LJI308 + morphine group (*n* = 3, *P* = 0.001, compared with morphine + DMSO group, one-way ANOVA), respectively (Fig. 4a). We also examined whether U0126 or LJI 308 alone had an influence on the expression of GR in primary cultured mPFC neurons. The results showed that U0126 or LJI 308 alone had no effect on GR expression in primary cultured mPFC neurons (U0126: 1.246 ± 0.132 and LJI 308: 0.874 ± 0.153, *n* = 3 in each group), compared with the control group (1.000 ± 0.046, *n* = 3, *P* = 0.1611, one-way ANOVA) (Supplementary data Fig. 3). We further investigated the effect of GR deletion on the phosphorylation of CREB induced by morphine. Primary cultured neurons were transfected with LV-GR-RNAi or LV-NC-RNAi and then treated with morphine for 72 h. Neurons were divided into four groups: LV-NC-RNAi group, LV-GR-RNAi group, morphine + LV-NC-RNAi group, and morphine + LV-GR-RNAi group. The results showed that GR deletion did not alter the upregulation of p-CREB induced by morphine (1.237 ± 0.073 vs 1.174 ± 0.058, *n* = 3, *P* = 0.097, one-way ANOVA) (Fig. 4b). These results suggest that mu receptor-ERK-p90RSK-CREB signaling pathway may mediate chronic morphine-induced increase in the expression of GR in the mPFC of rats.

### Chronic morphine-induced increase of GR in the mPFC plays an important role in conditioned context-induced retrieval of morphine withdrawal memory in rats

**Fig. 5:**
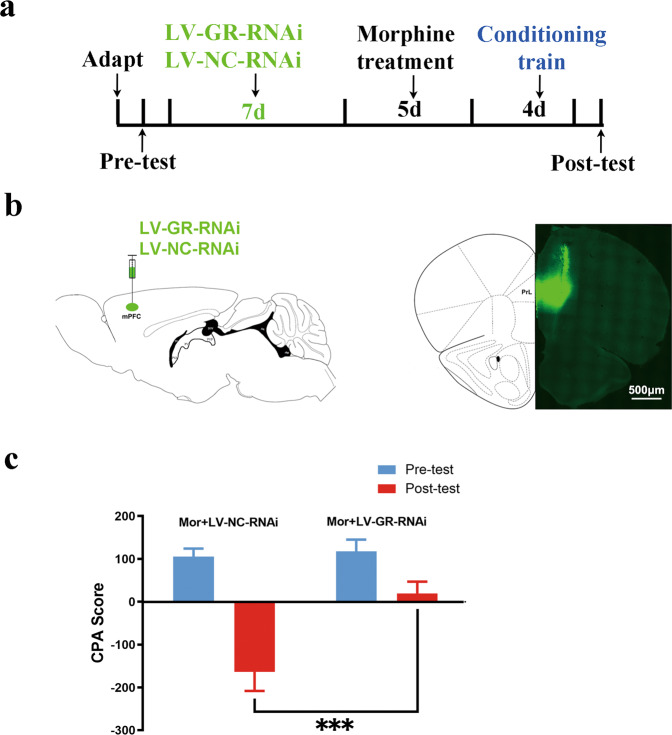
Effect of GR expression induced by chronic morphine treatment on the retrieval of contextual morphine withdrawal memory. **a** Experimental timeline for the CPA procedure. **b** Left: Diagram of virus injection site in mPFC. Right: Image of coronal brain slice showing the expression of LV virus in mPFC. Scale bar, 500 μm. **c** Average pre-test and post-test CPA scores in Morphine + LV-NC-RNAi and Morphine + LV-GR-RNAi group (two-way ANOVA, virus treatment factor, *F*_(1,38)_ = 9.996, *P* = 0.0031; test condition factor, *F*_(1,38)_ = 35.56, *P* < 0.0001; virus treatment x condition, *F*_(1,38)_ = 7.594, *P* = 0.0089; Bonferroni post hoc analysis). The post-test CPA scores in Morphine + LV-GR-RNAi was significantly lower than that of the morphine + LV-NC-RNAi group (two-way ANOVA, Bonferroni post hoc analysis, *t* = 4.184, ***P = 0.0003). Data are presented as mean ± SEM.

In our previous study, we demonstrated that (1) chronic morphine-induced increases in the expression of D1 receptors at presynaptic terminals coming from other structures to the BLA played an important role in conditioned context-induced retrieval of morphine withdrawal memory;[9] (2) the downregulation of miR-105 in neurons projecting from the mPFC to the BLA was the reason for chronic morphine-induced increases in the expression of D1 receptors at presynaptic terminals coming from mPFC to the BLA and the overexpression of miR-105 in the mPFC inhibited context-induced retrieval of morphine withdrawal memory [10]. In this study, we characterized intracellular mechanisms underlying chronic morphine-induced decrease in the expression of miR-105 after chronic morphine and found that GR in the mPFC played an important role in chronic morphine-induced decrease in the expression of miR-105 in the mPFC. Therefore, it was most likely that chronic morphine-induced increase in the expression of GR in the mPFC also played an important role in conditioned context-induced retrieval of morphine withdrawal memory. To test this hypothesis, we examined the influence of the inhibition of GR using RNAi method on CPA, which was a typical animal model of conditioned context-induced retrieval of morphine withdrawal memory [22]. The rats were divided into two groups: Morphine + LV-GR-RNAi group and Morphine + LV-NC-RNAi group. The CPA training process (Fig. 5a) was executed 7 days after the rats were injected with LV-GR-RNAi or LV-NC-RNAi into the mPFC (Fig. 5b) before chronic morphine treatment. The results showed the rats in the morphine + LV-NC-RNAi group exhibited a strong aversion to withdrawal-paired compartment, while the rats in the morphine + LV-GR-RNAi group had no significant aversive responses for the two compartments (two-way ANOVA, virus treatment factor, *F*_(1,38)_ = 9.996, *P* = 0.0031; test condition factor, *F*_(1,38)_ = 35.56, *P* < 0.0001; virus treatment x condition, *F*_(1,38)_ = 7.594, *P* = 0.0089; Bonferroni post hoc analysis). Average post-test CPA score of the morphine + LV-GR-RNAi group (18.88 ± 28.19 s, *n* = 11) was significantly lower than that of the morphine + LV-NC-RNAi group (−163.7 ± 44.49 s, *n* = 10; two-way ANOVA, Bonferroni post hoc analysis, *t* = 4.184, *P* = 0.0003, compared to the post-test CPA score in morphine + LV-GR-RNAi group) (Fig. 5c). This result suggests that chronic morphine-induced increase of GR in the mPFC plays an important role in conditioned context-induced retrieval of morphine withdrawal memory in rats.

## Discussion

Our previous study showed that chronic morphine significantly decreased miR-105 in the mPFC, which was closely related to conditioned context-induced retrieval of morphine withdrawal memory [10]. In this paper, we repeated this experiment and confirmed that chronic morphine indeed could decrease miR-105 in the mPFC. Moreover, here we examined the influence of chronic morphine on the expression of the precursor of miR-105 (pri-miR-105) in the mPFC and the result showed that chronic morphine could decrease the expression of pri-miR-105, suggesting that the decrease of the precursor of miR-105 might be the main reason of chronic morphine-induced decrease of miR-105 in the mPFC.

We further studied signaling pathways underlying chronic morphine-induced decrease of miR-105 in the mPFC. The classical signaling pathways underlying the analgesic effect of morphine is that the activation of opioid receptors leads to an inhibition of adenylate cyclase activity and a reduction of cAMP levels as well as a suppression of the activity of protein kinase A [23]. However, based on our present results, it appears that the signaling pathways after the activation of opioid receptors by morphine to induce addiction-related change in miR-105 are different to that of morphine-induced analgesia, that is, morphine decreases the expression of miR-105 in the mPFC via mu receptors-ERK-p90RSK-CREB signaling pathway.

It has been known that CREB is a transcription factor that binds to cAMP response element (CRE) as a dimer to activate transcription [3]. Therefore, the direct effect of CREB should not be able to inhibit the expression of miR-105. GR is a transcription factor that can bind to promoter regions to induce transrepression [19]. So, we propose a hypothesis that chronic morphine may decrease the expression of miR-105 in the mPFC by two kinds of transcription factors: CREB as the first transcription factor and GR as the second transcription factor. This hypothesis is confirmed by our present results. To our knowledge, this is the first report showing that morphine first activates one kind of transcription by CREB and then activates a negative transcription factor GR that inhibits the transcription of targeted gene to modulate the expression of genes.

In addition to be a transcription factor, GR is also the receptor of glucocorticoids (GCs). Previous studies showed that acutely administered morphine significantly increased the plasma levels of GCs via the action on the hypothalamo-pituitary-adrenocortical system [24, 25]. This increase of GCs plays a key role in mediating the biological effects of morphine, such as the development of behavioral sensitization [26], physical dependence [5], and reward learning [27–29]. However, Milanés et al. reported that in chronically morphine-treated rats, there was no significant change in plasma GCs levels [24]. This result combined with our present finding suggests that chronic morphine do not induce biological effects through increasing local GCs concentration, but may do it by enhancing the effect of GR as a negative transcription factor and this effect of GR may play an important role in the retrieval of drug withdrawal memory.

In conclusion, the present results suggest that two kinds of transcription factors, CREB as the first transcription factor and GR as the second transcription factor, mediate chronic morphine-induced decrease in miR-105 in the mPFC of rats and this pathway mediates conditioned context-induced retrieval of morphine withdrawal memory in rats.

## Supplementary information

supplementary data

all authors agreement email
