# Supplementary material for: Two kinds of transcription factors mediate chronic morphine-induced decrease in miR-105 in medial prefrontal cortex of rats
Source: Transl Psychiatry. 2022 Oct 31;12:458. doi: 10.1038/s41398-022-02222-3 (PMC9622915; doi:10.1038/s41398-022-02222-3)
Supplement: Supplementary file 1 — supplementary data [file 41398_2022_2222_MOESM1_ESM.docx]

**Supplementary data**

**The acute effect of morphine on miR-105 expression in rat mPFC and cultured mPFC neurons**

MiR-105 expression was detected by qRT-PCR in rat mPFC 2h after one dose morphine(10mg/kg) injected. The result showed that no significant change was observed in miR-105 expression in rat mPFC (1.066 ± 0.078, n=4, t=0.1165, *P*>0.05, compared with saline group 1.044 ± 0.173, n=4) (sFigure 1a). The mPFC neurons 2h treated by morphine (10μM) were collected to detect the miR-105 expression by qRT-PCR. Result showed the expression of miR-105 in the cultured mPFC neurons had no change (0.749±0.183, n=3, t=1.089, *P*>0.05, compared saline group 1.029±0.181, n=3) (sFigure 1b). Our results suggest morphine induced miR-105 expression inhibition is not an acute effect.


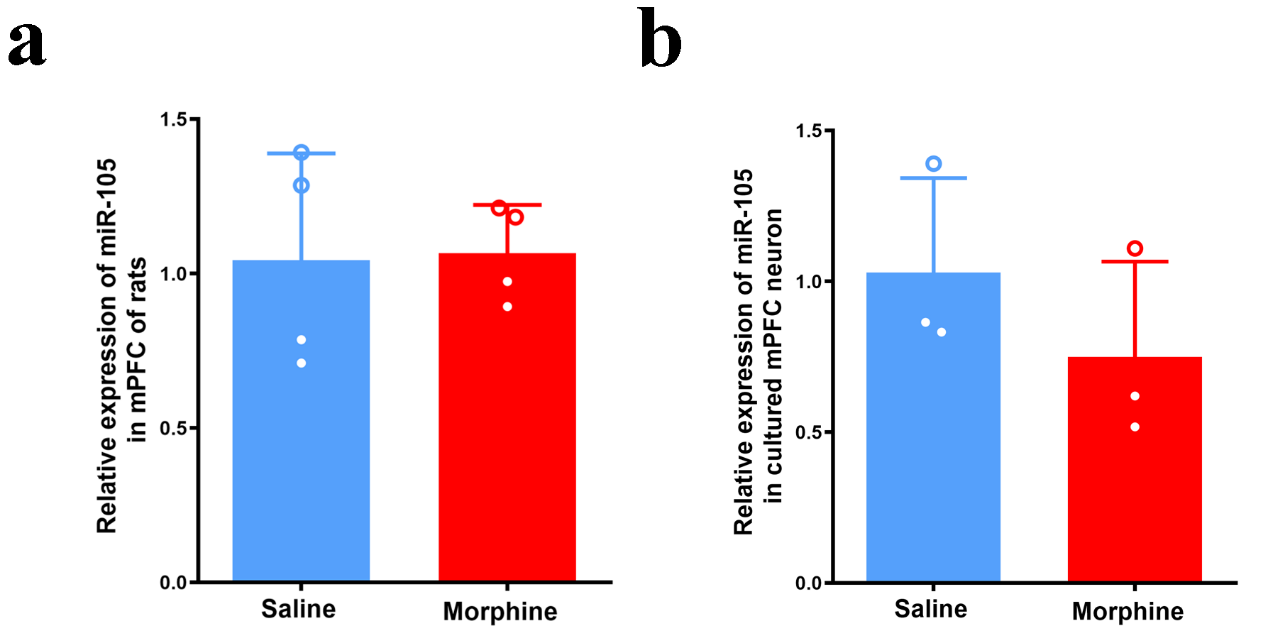
sFigure 1: The acute effect of morphine on miR-105 expression. a: miR-105 expression in rat mPFC 2h after one dose morphine (10 mg/kg) injection (n=3 in each group, unpaired two-tailed Student's t-test, *P*>0.05). b: miR-105 expression in primary cultured mPFC neurons 2h after morphine (10μM) treatment (n=3 in each group, unpaired two-tailed Student's t-test, *P*>0.05).

**The effect of naloxone on the expression of miR-105** **in cultured mPFC neurons**

The mPFC neurons were cultured for 5 days and then treated by naloxone (10μM) for 3 days. Result showed naloxone treatment had no significantly influence on miR-105 expression (unpaired t-test, 1.589 ± 0.161, n=3, *t*=2.315, *P*=0.082), compared with control group (1.029 ± 0.181, n=3) (sFigure 2)


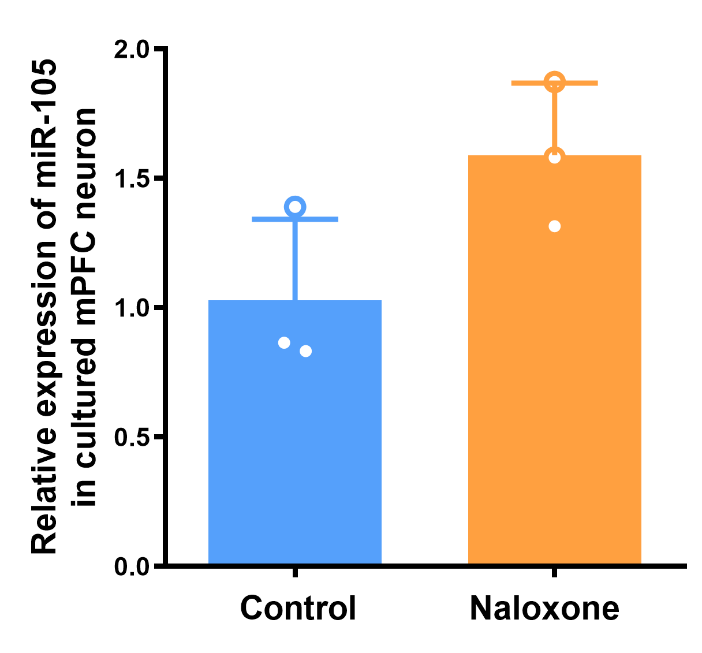


sFigure 2 The effect of naloxone(10μM) on miR-105 expression in primary cultured mPFC neurons (n=3 in each group, unpaired two-tailed Student's t-test, *P*>0.05).

**The effect of U0126 (10μM) or LJI-308 (1μM) on GR expression in primary cultured mPFC neurons**

The primary cultured neurons were treated by U0126(10μM) or LJI-308(1μM) for 3 days and the GR expression was detected by western blot. Results showed U0126 and LJI 308 had no effect on GR expression (1.000 ± 0.046,1.246 ± 0.132, 0.874 ± 0.153 in control group, U0126 group and LJI308 group, respectively; one-way ANOVA, *F*_(2,6)_=0.4316, *P*>0.05) (sFigure 2).


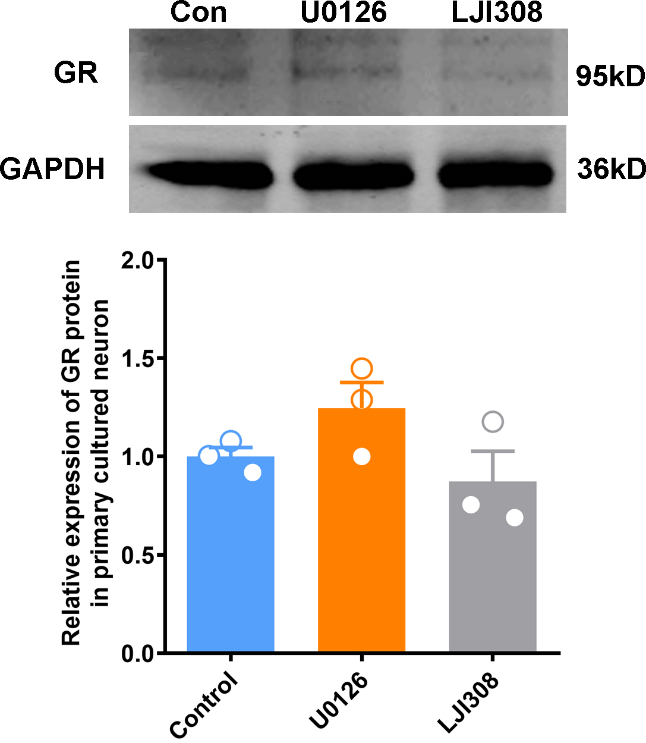


sFigure 3 The effect of U0126(10μM) or LJI-308(1μM) on GR expression in primary cultured mPFC neuron (one-way ANOVA, *F*_(2,6)_=0.4316, *P*>0.05).
