# Supplementary material for: Two kinds of transcription factors mediate chronic morphine-induced decrease in miR-105 in medial prefrontal cortex of rats
Source: Transl Psychiatry. 2022 Oct 31;12:458. doi: 10.1038/s41398-022-02222-3 (PMC9622915; doi:10.1038/s41398-022-02222-3)
Supplement: Supplementary file 2 — all authors agreement email [file 41398_2022_2222_MOESM2_ESM.pdf]

FUDAN UNIVERSITY

zheng ping <pzheng@shmu.edu.cn> 自助查询 - 锁屏 设置 | 帮助 | 退出 搜索邮件

欢迎页 收件箱 回复：Abut ...

收 信 写 信

回复 回复全部 转发 删除 来信分类 举报 标记为 移动到 更多

收件箱
草稿箱
已发送
其他文件夹 (53...
个人通讯录
会议与日程
邮箱中心
代收邮箱
来信分类
文件中心
文件中转站
个人网盘

时 间：2022年10月05日 17:41:33 (星期三)

收件人：zheng ping <pzheng@shmu.edu.cn>

Dear Prof. Zheng

I agree with the author change about the manuscript.

Junfangzh Zhang
发自我的荣耀手机

-------- 原始邮件 --------
发件人： zheng ping <pzheng@shmu.edu.cn>
日期： 2022年10月5日周三 下午1:52
收件人： 张俊芳 <junfangzh@126.com>
主 题： Abut manuscript author change

Dr Junfang Zhang,

Based on the contribution to the manuscript "Two kinds of transcription factors mediate chronic morphine-induced decrease in miR-105 in medial prefrontal cortex of rats", Mr Pan Yan will be changed to co-first author of this manuscript. Tne new list of the authors was: the author list will be change to Dr Junfang Zhang , Ms Xinli Guo , Mr Zhangyin Cai , Dr Yan Pan , Ms Hao Yang , Ms Yali Fu , Ms Zixuan Cao , Ms Yaxian Wen , Mr Chao Lei , Ms Chenshan Chu , Ms Yu Yuan , Dr Dongyang Cui , Mr Pengyu Gao , Professor Bin Lai, Professor Ping Zheng. Among it, Dr Junfang Zhang , Ms Xinli Guo , Mr Zhangyin Cai , Dr Yan Pan were the co-first author and Professor Lai Bin, Professor Ping Zheng were co-corresponding author. Do you agree to this change?

复旦大学 版权所有 © 2010 - 2013. 服务电话: 65643207 65643247 Email: urp@fudan.edu.cn

Re: Abut manuscript author change 发起会议 精简信息

发件人： 12472170... <1247217042@qq.com>
时 间： 2022年10月05日 14:59:21 (星期三)
收件人： 复旦大学郑平老师 <pzheng@shmu.edu.cn>

Yes, I agree to this change.

Best

Xinli Guo

---- Replied Message ----

From zheng ping<pzheng@shmu.edu.cn>
Date 10/05/2022 14:46
To 1247217042@qq.com<1247217042@qq.com>
Subject Abut manuscript author change

Ms Xinli Guo,

Based on the contribution to the manuscript "Two kinds of transcription factors mediate chronic morphine-induced decrease in miR-105 in medial prefrontal cortex of rats", Mr Pan Yan will be changed to co-first author of this manuscript. Tne new list of the authors was: the author list will be change to Dr Junfang Zhang , Ms Xinli Guo , Mr Zhangyin Cai , Dr Yan Pan , Ms Hao Yang , Ms Yali Fu , Ms Zixuan Cao , Ms Yaxian Wen , Mr Chao Lei , Ms Chenshan Chu , Ms Yu Yuan , Dr Dongyang Cui , Mr Pengyu Gao , Professor Bin Lai, Professor Ping Zheng. Among it, Dr Junfang Zhang , Ms Xinli Guo , Mr Zhangyin Cai , Dr Yan Pan were the co-first author and Professor Lai Bin, Professor Ping Zheng were co-corresponding author. Do you agree to this change?

>Best,

>Ping
>
>
>
>
>
>
>
>
>

复旦大学 版权所有 © 2010 - 2013. 服务电话: 65643207 65643247 Email: urp@fudan.edu.cn

Re:Abut manuscript author change 发起会议 精简信息

发件人: 复旦大学蔡张胤 <czybioscience@163.com>
时 间: 2022年10月05日 17:35:49 (星期三)
收件人: zheng ping <pzheng@shmu.edu.cn>

>Prof. Ping Zheng,
>I absolutely agree to this change.
>>Best,
>> Zhangyin Cai

At 2022-10-05 16:08:34, "zheng ping" <pzheng@shmu.edu.cn> wrote:
>Mr Zhangyin Cai ,
>
>Based on the contribution to the manuscript "Two kinds of transcription factors mediate chronic morphine-induced decrease in miR-105 in medial prefrontal cortex of rats", Mr Pan Yan will be changed to co-first author of this manuscript. Tne new list of the authors was: the author list will be change to Dr Junfang Zhang , Ms Xinli Guo , Mr Zhangyin Cai , Dr Yan Pan , Ms Hao Yang , Ms Yali Fu , Ms Zixuan Cao , Ms Yaxian Wen , Mr Chao Lei , Ms Chenshan Chu , Ms Yu Yuan , Dr Dongyang Cui , Mr Pengyu Gao , Professor Bin Lai, Professor Ping Zheng. Among it, Dr Junfang Zhang , Ms Xinli Guo , Mr Zhangyin Cai , Dr Yan Pan were the co-first author and Professor Lai Bin, Professor Ping Zheng were co-corresponding author. Do you agree to this change?
>
>>Best,

复旦大学 版权所有 © 2010 - 2013. 服务电话: 65643207 65643247 Email: urp@fudan.edu.cn

FUDAN UNIVERSITY
zheng ping <pzheng@shmu.edu.cn> 自助查询 - 锁屏 设置 | 帮助 | 退出 搜索邮件
欢迎页 收件箱 回复：Abut ... Re:Abut man... Re: Abut man... Re: Abut man... 回复：Abut ... Re: Abut m...
收 信 写 信
回复 回复全部 转发 删除 来信分类 举报 标记为 移动到 更多
收件箱
草稿箱
已发送
其他文件夹 (53...
个人通讯录
会议与日程
邮箱中心
代收邮箱
来信分类
文件中心
文件中转站
个人网盘

Re: Abut manuscript author change 发起会议 精简信息

发件人： 杨皓 <yh60021222@126.com>
时 间： 2022年10月05日 16:20:13 (星期三)
收件人： pzheng <pzheng@shmu.edu.cn>

Yes, I agree to that change. ----Hao Yang

杨 皓
复旦大学脑科学研究院神经生物学国家重点实验室
Tel：18301896102
地址：复旦大学上海医学院治道楼903
邮编：200032
Email：16111520009@fudan.edu.cn

---- Replied Message ----

From zheng ping<pzheng@shmu.edu.cn>
Date 10/05/2022 14:39
To yh60021222@126.com<yh60021222@126.com>
Subject Abut manuscript author change

Ms Hao Yang,

Based on the contribution to the manuscript "Two kinds of transcription factors mediate chronic morphine-induced decrease in miR-105 in medial prefrontal cortex of rats", Mr Pan Yan will be changed to co-first author of this manuscript. Tne new list of the authors was: the author list will be change to Dr Junfang Zhang , Ms Xinli Guo , Mr Zhangyin Cai , Dr Yan Pan , Ms Hao Yang , Ms Yali Fu , Ms Zixuan Cao , Ms Yaxian Wen , Mr Chao Lei , Ms Chenshan Chu , Ms Yu Yuan , Dr Dongyang Cui , Mr Pengyu [illegible]

复旦大学 版权所有 © 2010 - 2013. 服务电话: 65643207 65643247 Email: urp@fudan.edu.cn

Re: Abut manuscript author change

发起会议 精简信息

发件人: fu486912... <fu48691201@126.com>
时 间: 2022年10月05日 16:21:26 (星期三)
收件人: zheng ping <pzheng@shmu.edu.cn>

Yes, I agree to this change.

Yali Fu

---Original---

From: "zheng ping"<pzheng@shmu.edu.cn>
Date: Wed, Oct 5, 2022 14:44 PM
To: "fu48691201"<fu48691201@126.com>;
Subject: Abut manuscript author change

Ms Yali Fu ,

Based on the contribution to the manuscript "Two kinds of transcription factors mediate chronic morphine-induced decrease in miR-105 in medial prefrontal cortex of rats", Mr Pan Yan will be changed to co-first author of this manuscript. Tne new list of the authors was: the author list will be change to Dr Junfang Zhang , Ms Xinli Guo , Mr Zhangyin Cai , Dr Yan Pan , Ms Hao Yang , Ms Yali Fu , Ms Zixuan Cao , Ms Yaxian Wen , Mr Chao Lei , Ms Chenshan Chu , Ms Yu Yuan , Dr Dongyang Cui , Mr Pengyu Gao , Professor Bin Lai, Professor Ping Zheng. Among it, Dr Junfang Zhang , Ms Xinli Guo , Mr Zhangyin Cai , Dr Yan Pan were the co-first author and Professor Lai Bin, Professor Ping Zheng were co-corresponding author. Do you agree to this change?

>Best,

>Ping
>
>
>

复旦大学 版权所有 © 2010 - 2013. 服务电话: 65643207 65643247 Email: urp@fudan.edu.cn

Re: Abut manuscript author change 发起会议 精简信息

发件人： 476167079 <476167079@qq.com>
时 间： 2022年10月05日 16:21:14 (星期三)
收件人： zheng ping <pzheng@shmu.edu.cn>

Yes, I agree.

Zixuan Cao

---Original---

From: "zheng ping"<pzheng@shmu.edu.cn>
Date: Wed, Oct 5, 2022 15:07 PM
To: "476167079"<476167079@qq.com>;
Subject: Abut manuscript author change

Ms Zixuan Cao,

Based on the contribution to the manuscript "Two kinds of transcription factors mediate chronic morphine-induced decrease in miR-105 in medial prefrontal cortex of rats", Mr Pan Yan will be changed to co-first author of this manuscript. Tne new list of the authors was: the author list will be change to Dr Junfang Zhang , Ms Xinli Guo , Mr Zhangyin Cai , Dr Yan Pan , Ms Hao Yang , Ms Yali Fu , Ms Zixuan Cao , Ms Yaxian Wen , Mr Chao Lei , Ms Chenshan Chu , Ms Yu Yuan , Dr Dongyang Cui , Mr Pengyu Gao , Professor Bin Lai, Professor Ping Zheng. Among it, Dr Junfang Zhang , Ms Xinli Guo , Mr Zhangyin Cai , Dr Yan Pan were the co-first author and Professor Lai Bin, Professor Ping Zheng were co-corresponding author. Do you agree to this change?

>Best,

>Ping
>
>
>
>
>
>

复旦大学 版权所有 © 2010 - 2013. 服务电话: 65643207 65643247 Email: urp@fudan.edu.cn

FUDAN UNIVERSITY

zheng ping <pzheng@shmu.edu.cn> 自助查询 - 锁屏 设置 | 帮助 | 退出 搜索邮件

欢迎页 收件箱 回复: Abut ... Re:Abut ma... Re: Abut ma... Re: Abut ma... 回复: Abut ... Re: Abut ma... Re:Abut ma...

收信 写信

回复 回复全部 转发 删除 来信分类 举报 标记为 移动到 更多

收件箱
草稿箱
已发送
其他文件夹 (53...
个人通讯录
会议与日程
邮箱中心
代收邮箱
来信分类
文件中心
文件中转站
个人网盘

Re: Abut manuscript author change 发起会议 精简信息

发件人: 文不文 <502480082@qq.com>
时 间: 2022年10月05日 15:11:11 (星期三)
收件人: zheng ping <pzheng@shmu.edu.cn>

Yes, I agree.

Best,

Yaxian Wen

发自我的iPhone

------------------ Original ------------------

From: zheng ping <pzheng@shmu.edu.cn>
Date: Wed, Oct 5, 2022 2:54 PM
To: 502480082 <502480082@qq.com>
Subject: Re: Abut manuscript author change

Ms Yaxian Wen,

Based on the contribution to the manuscript "Two kinds of transcription factors mediate chronic morphine-induced decrease in miR-105 in medial prefrontal cortex of rats", Mr Pan Yan will be changed to co-first author of this manuscript. Tne new list of the authors was: the author list will be change to Dr Junfang Zhang , Ms Xinli Guo , Mr Zhangyin Cai , Dr Yan Pan , Ms Hao Yang , Ms Yali Fu , Ms Zixuan Cao , Ms Yaxian Wen , Mr Chao Lei , Ms Chenshan Chu , Ms Yu Yuan , Dr Dongyang Cui , Mr Pengyu Gao , Professor Bin Lai, Professor Ping Zheng. Among it, Dr Junfang Zhang , Ms Xinli Guo , Mr Zhangyin Cai , Dr Yan Pan were the co-first author and Professor Lai Bin, Professor Ping Zheng were co-corresponding author. Do you agree to this change?

>Best,

复旦大学 版权所有 © 2010 - 2013. 服务电话: 65643207 65643247 Email: urp@fudan.edu.cn

FUDAN UNIVERSITY
zheng ping <pzheng@shmu.edu.cn> 自助查询 - 锁屏 设置 | 帮助 | 退出 搜索邮件
欢迎页 收件箱 Re: Abut man... Re: Abut m...
收 信 写 信
回复 回复全部 转发 删除 来信分类 举报 标记为 移动到 更多
收件箱
草稿箱
已发送
其他文件夹 (53...
个人通讯录
会议与日程
邮箱中心
代收邮箱
来信分类
文件中心
文件中转站
个人网盘

Re: Abut manuscript author change 发起会议 精简信息

发件人: uuuleichao <uuuleichao@163.com>
时 间: 2022年10月05日 16:24:54 (星期三)
收件人: zheng ping <pzheng@shmu.edu.cn>

Yes, I agree.

Chao Lei

---- Replied Message ----

From zheng ping<pzheng@shmu.edu.cn>
Date 10/05/2022 15:05
To uuuleichao<uuuleichao@163.com>
Subject Abut manuscript author change

Mr Chao Lei,

Based on the contribution to the manuscript "Two kinds of transcription factors mediate chronic morphine-induced decrease in miR-105 in medial prefrontal cortex of rats", Mr Pan Yan will be changed to co-first author of this manuscript. Tne new list of the authors was: the author list will be change to Dr Junfang Zhang , Ms Xinli Guo , Mr Zhangyin Cai , Dr Yan Pan , Ms Hao Yang , Ms Yali Fu , Ms Zixuan Cao , Ms Yaxian Wen , Mr Chao Lei , Ms Chenshan Chu , Ms Yu Yuan , Dr Dongyang Cui , Mr Pengyu Gao , Professor Bin Lai, Professor Ping Zheng. Among it, Dr Junfang Zhang , Ms Xinli Guo , Mr Zhangyin Cai , Dr Yan Pan were the co-first author and Professor Lai Bin, Professor Ping Zheng were co-corresponding author. Do you agree to this change?

>Best,

>Ping
>
>
>
>

复旦大学 版权所有 © 2010 - 2013. 服务电话: 65643207 65643247 Email: urp@fudan.edu.cn

FUDAN UNIVERSITY

zheng ping <pzheng@shmu.edu.cn> 自助查询 - 锁屏 设置 | 帮助 | 退出 搜索邮件

欢迎页 收件箱 回复：Abut ...

收 信 写 信

回复 回复全部 转发 删除 来信分类 举报 标记为 移动到 更多

收件箱
草稿箱
已发送
其他文件夹 (53...
个人通讯录
会议与日程
邮箱中心
代收邮箱
来信分类
文件中心
文件中转站
个人网盘

回复：Abut manuscript author change

发起会议 精简信息

发件人: 储辰珊 <shanshan3213@vip.qq.com>
时 间: 2022年10月05日 16:31:03 (星期三)
收件人: zheng ping <pzheng@shmu.edu.cn>

Yes, I agree.

Chenshan Chu

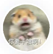

458303213@qq.com

------------------ 原始邮件 ------------------

发件人: "zheng ping" <pzheng@shmu.edu.cn>;
发送时间: 2022年10月5日(星期三) 下午2:56
收件人: "储辰珊"<shanshan3213@vip.qq.com>;
主题: Abut manuscript author change

Ms Chenshan Chu,

Based on the contribution to the manuscript "Two kinds of transcription factors mediate chronic morphine-induced decrease in miR-105 in medial prefrontal cortex of rats", Mr Pan Yan will be changed to co-first author of this manuscript. Tne new list of the authors was: the author list will be change to Dr Junfang Zhang , Ms Xinli Guo , Mr Zhangyin Cai , Dr Yan Pan , Ms Hao Yang , Ms Yali Fu , Ms Zixuan Cao , Ms Yaxian Wen , Mr Chao Lei , Ms Chenshan Chu , Ms Yu Yuan , Dr Dongyang Cui , Mr Pengyu Gao , Professor Bin Lai, Professor Ping Zheng. Among it, Dr Junfang Zhang , Ms Xinli Guo , Mr Zhangyin Cai , Dr Yan Pan were the co-first author and Professor Lai Bin, Professor Ping Zheng were co-corresponding author. Do you agree to this change?

>Best,

>Ping
>
>

复旦大学 版权所有 © 2010 - 2013. 服务电话: 65643207 65643247 Email: urp@fudan.edu.cn

FUDAN UNIVERSITY

zheng ping <pzheng@shmu.edu.cn> 自助查询 - 锁屏 设置 | 帮助 | 退出 搜索邮件

欢迎页 收件箱 回复：Abut ... Re:Abut man... 回复：Abut ... Re: Abut man... Re: Abut man... re:Abut ma...

收 信 写 信

回复 回复全部 转发 删除 来信分类 举报 标记为 移动到 更多

收件箱
草稿箱
已发送
其他文件夹 (53...
个人通讯录
会议与日程
邮箱中心
代收邮箱
来信分类
文件中心
文件中转站
个人网盘

re:Abut manuscript author change 发起会议 精简信息

发件人: 1253409335 <1253409335@qq.com>
时 间: 2022年10月05日 15:02:52 (星期三)
收件人: pzheng <pzheng@shmu.edu.cn>

Yes,I agree!

------------------ 原始邮件 ------------------

发件人: "zheng ping";
发件时间: 2022-10-05 14:36
收件人: "1253409335"<1253409335@qq.com>;
主题: Abut manuscript author change

Ms Yu Yuan,

Based on the contribution to the manuscript "Two kinds of transcription factors mediate chronic morphine-induced decrease in miR-105 in medial prefrontal cortex of rats", Mr Pan Yan will be changed to co-first author of this manuscript. Tne new list of the authors was: the author list will be change to Dr Junfang Zhang , Ms Xinli Guo , Mr Zhangyin Cai , Dr Yan Pan , Ms Hao Yang , Ms Yali Fu , Ms Zixuan Cao , Ms Yaxian Wen , Mr Chao Lei , Ms Chenshan Chu , Ms Yu Yuan , Dr Dongyang Cui , Mr Pengyu Gao , Professor Bin Lai, Professor Ping Zheng. Among it, Dr Junfang Zhang , Ms Xinli Guo , Mr Zhangyin Cai , Dr Yan Pan were the co-first author and Professor Lai Bin, Professor Ping Zheng were co-corresponding author. Do you agree to this change?
>
> Best,
>
> Ping
>

复旦大学 版权所有 © 2010 - 2013. 服务电话: 65643207 65643247 Email: urp@fudan.edu.cn

Re: Abut manuscript author change 发起会议 精简信息

发件人: 崔冬阳 <dongyangcui@unixell.com>
时 间: 2022年10月05日 15:05:32 (星期三)
收件人: zheng ping <pzheng@shmu.edu.cn>

Yes, I agree to this change.

Best

Dongyang Cui

发自我的企业微信

On Wed,Oct 5,2022 2:47 PM zheng ping <pzheng@shmu.edu.cn> wrote:

> Dr Dongyang Cui,
>
> Based on the contribution to the manuscript "Two kinds of transcription factors mediate chronic morphine-induced decrease in miR-105 in medial prefrontal cortex of rats", Mr Pan Yan will be changed to co-first author of this manuscript. Tne new list of the authors was: the author list will be change to Dr Junfang Zhang , Ms Xinli Guo , Mr Zhangyin Cai , Dr Yan Pan , Ms Hao Yang , Ms Yali Fu , Ms Zixuan Cao , Ms Yaxian Wen , Mr Chao Lei , Ms Chenshan Chu , Ms Yu Yuan , Dr Dongyang Cui , Mr Pengyu Gao , Professor Bin Lai, Professor Ping Zheng. Among it, Dr Junfang Zhang , Ms Xinli Guo , Mr Zhangyin Cai , Dr Yan Pan were the co-first author and Professor Lai Bin, Professor Ping Zheng were co-corresponding author. Do you agree to this change?
>
> >Best,
>
> >Ping
> >

复旦大学 版权所有 © 2010 - 2013. 服务电话: 65643207 65643247 Email: urp@fudan.edu.cn

Re: Abut manuscript author change 发起会议 精简信息

发件人: gpy18800... <gpy18800...@163.com>
时 间: 2022年10月05日 15:10:33 (星期三)
收件人: zheng ping <pzheng@shmu.edu.cn>

Yes, I agree.

---- Replied Message ----

From zheng ping<pzheng@shmu.edu.cn>
Date 10/05/2022 15:09
To gpy18800350939<gpy18800350939@163.com>
Subject Abut manuscript author change

Mr Pengyu Gao,

Based on the contribution to the manuscript "Two kinds of transcription factors mediate chronic morphine-induced decrease in miR-105 in medial prefrontal cortex of rats", Mr Pan Yan will be changed to co-first author of this manuscript. Tne new list of the authors was: the author list will be change to Dr Junfang Zhang , Ms Xinli Guo , Mr Zhangyin Cai , Dr Yan Pan , Ms Hao Yang , Ms Yali Fu , Ms Zixuan Cao , Ms Yaxian Wen , Mr Chao Lei , Ms Chenshan Chu , Ms Yu Yuan , Dr Dongyang Cui , Mr Pengyu Gao , Professor Bin Lai, Professor Ping Zheng. Among it, Dr Junfang Zhang , Ms Xinli Guo , Mr Zhangyin Cai , Dr Yan Pan were the co-first author and Professor Lai Bin, Professor Ping Zheng were co-corresponding author. Do you agree to this change?

>Best,

>Ping
>
>
>
>
>
>

复旦大学 版权所有 © 2010 - 2013. 服务电话: 65643207 65643247 Email: urp@fudan.edu.cn

回复：Abut manuscript author change 发起会议 精简信息

发件人： 来滨 <laibinglaibing@qq.com>
时 间： 2022年10月05日 17:30:46 (星期三)
收件人： zheng ping <pzheng@shmu.edu.cn>

Yes， I agree!

best!

Bin Lai

来滨
laibinglaibing@qq.com

------------------ 原始邮件 ------------------
发件人： "pzheng"<pzheng@shmu.edu.cn>;
发送时间： 2022年10月5日(星期三) 下午2:42
收件人： "来滨"<laibinglaibing@qq.com>;
主题： Abut manuscript author change

Professor Bin Lai

Based on the contribution to the manuscript "Two kinds of transcription factors mediate chronic morphine-induced decrease in miR-105 in medial prefrontal cortex of rats", Mr Pan Yan will be changed to co-first author of this manuscript. Tne new list of the authors was: the author list will be change to Dr Junfang Zhang , Ms Xinli Guo , Mr Zhangyin Cai , Dr Yan Pan , Ms Hao Yang , Ms Yali Fu , Ms Zixuan Cao , Ms Yaxian Wen , Mr Chao Lei , Ms Chenshan Chu , Ms Yu Yuan , Dr Dongyang Cui , Mr Pengyu Gao , Professor Bin Lai, Professor Ping Zheng. Among it, Dr Junfang Zhang , Ms Xinli Guo , Mr Zhangyin Cai , Dr Yan Pan were the co-first author and Professor Lai Bin, Professor Ping Zheng were co-corresponding author. Do you agree to this change?

复旦大学 版权所有 © 2010 - 2013. 服务电话: 65643207 65643247 Email: urp@fudan.edu.cn
